# Electrochemical DNA Biosensor That Detects Early Celiac Disease Autoantibodies

**DOI:** 10.3390/s21082671

**Published:** 2021-04-10

**Authors:** Anna B. N. Nguyen, Marcos Maldonado, Dylan Poch, Tyler Sodia, Andrew Smith, Teisha J. Rowland, Andrew J. Bonham

**Affiliations:** 1 Biomolecular Sciences and Engineering Program, University of California, Santa Barbara, Santa Barbara, CA 93106, USA; anna.nguyen@ucsb.edu; 2 Department of Chemistry & Biochemistry, Metropolitan State University of Denver, Denver, CO 80204, USA; marcos.an.maldonado@gmail.com (M.M.); dpoch@msudenver.edu (D.P.); tsodia@mines.edu (T.S.); asmit295@msudenver.edu (A.S.); 3 Department of Molecular, Cellular, and Developmental Biology, University of Colorado, Boulder, CO 80309, USA; teisha.rowland@colorado.edu

**Keywords:** celiac disease, celiac disease autoantibodies, E-DNA-based biosensors, biosensor, celiac disease autoantibody epitope, celiac disease diagnostics

## Abstract

Although it is estimated that more than one million Americans have celiac disease (CD), it remains challenging to diagnose. CD, an autoimmune and inflammatory response following the ingestion of gluten-containing foods, has symptoms overlapping with other diseases and requires invasive diagnostics. The gold standard for CD diagnosis involves serologic blood tests followed by invasive confirmatory biopsies. Here, we propose a less invasive method using an electrochemical DNA (E-DNA) biosensor for CD-specific autoantibodies (AABs) circulating in blood. In our approach, CD-specific AABs bind a synthetic neoepitope, causing a conformational change in the biosensor, as well as a change in the environment of an attached redox reporter, producing a measurable current reduction. We assessed the biosensor’s ability to detect CD-specific patient-derived AABs in physiological buffer as well as buffer supplemented with bovine serum. Our biosensor was able to detect AABs in a dose-dependent manner; increased signal change correlated with increased AAB concentration with an apparent dissociation constant of 0.09 ± 0.03 units/mL of AABs. Furthermore, we found our biosensor to be target-specific, with minimal off-target binding of multiple unrelated biomarkers. Future efforts aimed at increasing sensitivity in complex media may build upon the biosensor design presented here to further improve CD AAB detection and CD diagnostic tools.

## 1. Introduction

Celiac disease (CD) prevalence has increased over the past few decades [1], comprising an estimated 1% of the population today [1], although many remain undiagnosed due to an overlap of symptoms with other diseases and the invasive diagnostics required [2]. While CD cases are thought to have a singular cause—an immune response against gliadin [3], the water-insoluble component of gluten—symptoms are varied. Common symptoms include abdominal pain, diarrhea, villous atrophy, and weight loss, as well as extraintestinal symptoms including anemia, fatigue, neurological disorders, and mood disorders [4]. If left unmanaged, CD can cause an increased risk of other long-term complications including autoimmune disorders, female infertility, osteoporosis, thyroid disease, lymphocytic colitis, hyposplenism, and a general deterioration in quality of life [4]. Further complicating diagnoses is “silent CD”; afflicted individuals are asymptomatic but can still develop long-term complications [5]. To prevent this, and effectively treat symptoms caused by CD, early and accurate CD detection is crucial. However, CD is challenging to diagnose based on symptoms alone due to the wide range of possible symptoms and complications. The gold standard for diagnosing CD involves performing an invasive small intestine biopsy followed by histological analysis and positive identification of villi atrophy and mucosal lesions. These features are usually irregular; therefore, at least four to six biopsy samples are recommended [4]. Understandably, less invasive diagnostics are desirable.

To develop less invasive CD diagnostics, targeting CD-specific autoantibodies (AABs) present in patient serological samples is a promising research avenue, although improved specificity could be beneficial. In individuals with CD, an autoimmune response results in the body producing AABs, such as immunoglobin A (IgA) and immunoglobin G (IgG), against tissue transglutaminase (tTG), deamidated gliadin (GDP), and endomysial antibodies (EMAs). Enzyme-linked immunosorbent assay (ELISA)-based approaches have been developed to detect these AABs [6,7]. However, the presence of AABs against these targets does not necessarily indicate that an individual has CD. A new, more promising target for increased CD-detection specificity is a neoepitope that forms when undigested gliadin fragments crosslink with tTG. This neoepitope, which stimulates T-cells to upregulate anti-tTG AABs [3], has been reproduced as a synthetic short peptide that can be detected using CD-specific IgG AABs in ELISAs [8]; it remains to be reported whether this synthetic neoepitope is also recognized by IgA AABs, the preferred CD biomarker [4]. This is the preferred CD biomarker; therefore, recognition by IgA AABs of this synthetic neoepitope would likely result in increased diagnostic efficacy.

Electrochemical biosensors are promising and inexpensive rapid diagnostic tools for the sensitive and specific detection of CD-specific AABs and epitopes, with the potential to be used for point-of-care diagnostics [9]. These biosensors employ a probe containing biological recognition molecules to enable the detection of numerous targets [10,11], including detecting proteins at medically relevant nanomolar and picomolar concentrations [12,13]. A major subset of electrochemical biosensors is electrochemical DNA (E-DNA) biosensors, which utilize DNA oligomers covalently bound to an electrode surface. These oligomers typically undergo a conformational change in the target-bound, versus unbound, state. By attaching a redox-active molecule (e.g., methylene blue) to the oligomer, this conformational change alters the electron transfer kinetics with the electrode surface, producing a measurable change in current [12]. These E-DNA biosensors can reliably quantify antibody concentrations [14,15], detect target immune rejection marker proteins [15], detect ricin and botulism [16], and measure chemotherapeutics in vivo in real-time [17]. Furthermore, because E-DNA biosensors can detect biological molecules continuously and in real-time, they hold great promise for clinical use, where they could potentially reduce the time between the onset of symptoms and appropriate medical intervention.

**Figure 1:**
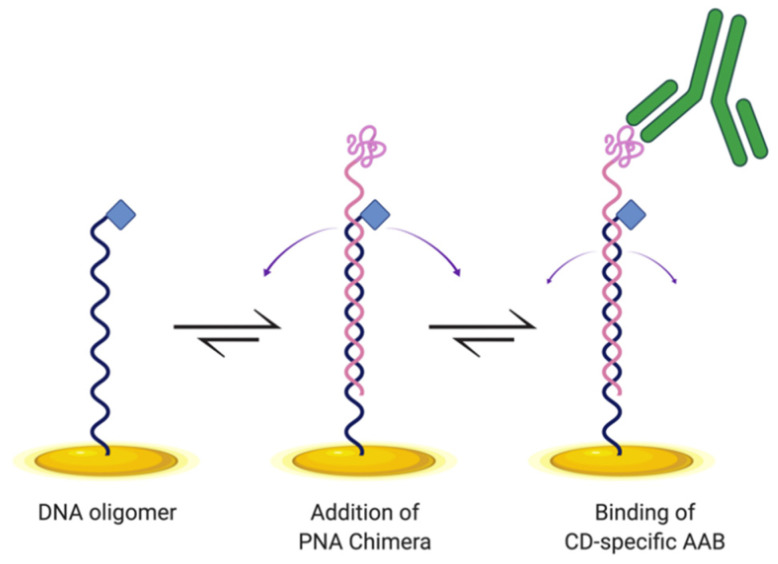
Schematic of E-DNA biosensor design for detecting celiac disease (CD) autoantibodies (AABs). DNA oligomer (black) is anchored to a gold electrode surface (gold) via a thiol–gold coordination bond, with methylene blue (blue diamond) functioning as a redox reporter. The DNA oligomer base-pairs to a peptide nucleic acid (PNA) chimera, which includes the gliadin-tTG synthetic neoepitope on the distal end (pink), and displays conformational flexibility (purple arrows) to enable electron transfer. Upon binding of the neoepitope by CD-specific AABs (green) present in a sample, the E-DNA biosensor is expected to undergo conformational change with reduced dynamics (smaller purple arrows), causing a reduction in peak current. Created with BioRender.com (accessed on 7 March 2021).

Here, we designed and tested an E-DNA biosensor for detecting CD AABs (Figure 1). This biosensor is similar in design to an E-DNA biosensor previously developed to detect the cytokine IP-10 [15]. The construct here hybridizes a DNA oligonucleotide with a complementary peptide nucleic acid (PNA) chimera, which is specifically a PNA oligomer (synthetic DNA mimic with peptide backbone) covalently distally bound to the gliadin-tTG synthetic neoepitope. We incorporated this neoepitope to enable biosensor binding of CD-specific AABs. To anchor the construct, a DNA oligomer includes a proximal modification enabling thiol-bonding to the gold electrode surface. To produce measurable changes in current, the anchoring DNA oligomer is distally modified to allow attachment of methylene blue to serve as the redox reporter. Here, we demonstrate that this E-DNA biosensor design is capable of detecting AABs in a dose-dependent manner, with increased signal correlating with increased AAB concentration, with minimal off-target binding, making it a promising basis for development as a novel diagnostic tool.

## 2. Materials and Methods

Unless otherwise noted, all chemicals were used as purchased from Sigma Aldrich, St. Louis, MO, USA.

### 2.1. Biosensor Design and Preparation

The E-DNA biosensor consisted of a gold screen-printed electrode (SPE) attached (via a thiol–gold coordination bond) to a DNA oligomer, the latter of which contained a distally attached redox reporter (i.e., 5′ attached methylene blue) and base-paired to a PNA chimera, a PNA oligomer covalently bound to a gliadin-tTG synthetic neoepitope. The SPE (Pine Research Instrumentation, Durham, NC, USA) consisted of a screen-printed system with a ceramic backing and three electrodes: 2.0 mm diameter gold working electrode, surrounding gold counter electrode, and a Ag/AgCl reference electrode. The total working area of the electrodes could be covered by 50 μL of buffer solution. SPEs were electrochemically cleaned as previously described, with minor modifications [18]. Specifically, SPEs were cleaned to remove organic surface contamination by voltametric scans in 0.5 M NaOH, 0.5 M H_2_SO_4_, and 0.1 M H_2_SO_4_/0.1 M KCl [18]. The DNA oligomer sequence was 5′-thiol-GCA GTA ACA AGA ATA AAA CGC CAC TGC-methylene blue-3′ and was synthesized and purified via reverse-phase HPLC (Integrated DNA Technologies (IDT), Coralville, IA, USA). The PNA chimera was designed and commercially prepared as follows (Panagene, Daejeon, South Korea): the PNA oligomer sequence was NEOEPITOPE–O–cag tgg cgt ttt att ctt gtt act g–CONH_2_, where lowercase letters are nucleosides, and the cross-linked dipeptide gliadin-tTG synthetic neoepitope sequence was DCLTESNLIK(N3)VR-, where N3 denotes an azide group, which was reacted with Ac-PQP(bpg)LPYPQ-CONH2, an acetylated peptide where bpg denotes a bihomopropargylglycine, the attachment site to the azide, creating the cross-linked neo-epitope.

To prepare our biosensor, the cleaned SPE was coated with a self-assembled monolayer of thiol–gold-bound DNA oligomers for 1 h and then backfilled with 1-mercapto-6-hexanol (MCH, Sigma Aldrich) [18]. All steps were performed at room temperature. To enable efficient thiol–gold bonding, the DNA was incubated for 1 h with molar excess tris (2-carboxyethyl) phosphine to reduce the methylene blue and thiol modifications. To allow complementary base-pairing of the PNA chimera to the DNA oligomer, SPEs with attached DNA oligomers were immersed for 1 h in binding buffer (1 × phosphate-buffered saline (PBS), consisting of 137 mM NaCl, 2.7 mM KCl, 10 mM Na_2_HPO_4_, 1.8 mM KH_2_PO_4_, pH 7.4, supplemented with 1 mM MgCl_2_ and 0.05% Tween 20) and then incubated with PNA chimera (9 µM) with binding buffer for 1 h. Attachment of the DNA oligomer and hybridization of the PNA chimera was confirmed via square-wave voltametric (SWV) analysis.

### 2.2. Electrochemical and Control Parameters

CD IgA AABs used were AESCQC Pool 4 (>100 units/mL anti-tTG IgA, 20 units/mL anti-Glia IgA, and >30 units/mL anti-Glia IgG) (AESKU.DIAGNOSTICS, Wendelsheim, Germany). This antibody mixture was the only one commercially available that included anti-tTG IgA antibodies; our biosensor was designed to only test the anti-tTG antibodies. CD AABs were titrated in binding buffer alone or binding buffer supplemented with 10% bovine serum (Sigma Aldrich). AAB titrations and off-target trials were all performed using a WaveNano Potentiostat System (Pine Research Instrumentation, Durham, NC, USA). During binding assays, between testing each solution the electrode was rinsed with binding buffer and then equilibrated with the new solution for 15 min. For serum trials, our PBS-based binding buffer was supplemented with adult bovine serum (10%, Sigma Aldrich, St. Louis, MO, USA). Biosensor function was interrogated using SWV from −0.5 V to −0.1 V versus Ag/AgCl, using an amplitude of 50 mV, potential step size of 5 mV, and frequency of 10 Hz. Post-experimental analysis was conducted using our previously published program SWVAnyPeakFinder [19] (code available at https://github.com/Paradoxdruid/SWVAnyPeakFinder, accessed on 1 December 2020) and graphs were prepared using PRISM 8 (GraphPad, San Diego, CA, USA, version 8.4.0). Apparent dissociation constants were obtained using PRISM 8 with a nonlinear, three-parameter dose–response model.

## 3. Results

**Figure 2:**
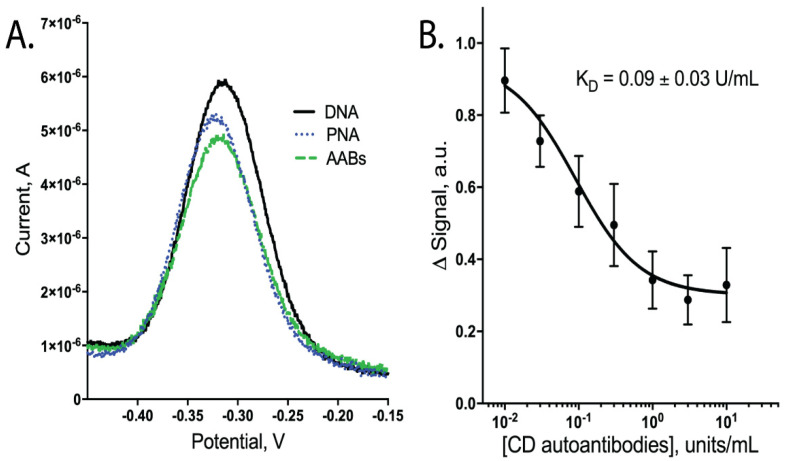
Binding performance of biosensors when tested with CD AABs in binding buffer (1× PBS with 1 mM MgCl2, 0.05% Tween 20, pH 7.4). (**A**) Square-wave voltammogram showing a decrease in peak current; signal in (**B**) calculated by change in peak height following PNA binding vs. addition of AABs. (**B**) Dose-dependent curve of signal change (arbitrary units) vs. CD AAB concentration with apparent dissociation constant (K_D_) of 0.09 ± 0.03 units/mL (*n* = 6; errors bars are SEM).

To detect CD AABs, we designed an E-DNA biosensor that changed conformation upon binding of the gliadin-tTG synthetic neoepitope by CD AABs present in a sample, resulting in a detectable decrease in current (Figure 1). In our design, a DNA oligomer, anchored to a gold electrode surface, hybridizes with a PNA chimera containing the neoepitope. This synthetic neoepitope mimics a neoepitope formed when undigested gliadin fragments crosslink with tTG; this neoepitope has been shown to stimulate T cells to upregulate anti-tTG AABs [3]. The neoepitope specifically used here is detectable in ELISAs when using CD-specific IgG AABs [8]. Therefore, upon exposure to a sample with these AABs, our biosensor system is expected to have its synthetic neoepitope recognized and bound by the AABs (Figure 1). This binding is predicted to generate a conformational change in the hybridized DNA oligomer. This oligomer contains a distal methylene blue redox reporter, which transfers electrons with the gold surface; therefore, this conformational change causes a peak current reduction readily observable using square-wave voltammetry (SWV) (Figure 2A, data available in Supplemental Table S1).

**Figure 3:**
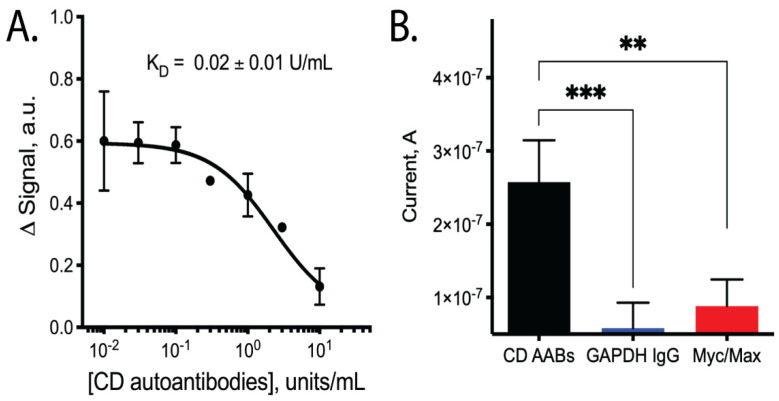
Binding performance of biosensors when tested with CD AABs in binding buffer supplemented with bovine serum and when tested with off-target antibodies. (**A**) Dose-dependent curve of signal change (arbitrary units) vs. CD AAB concentration when tested in binding buffer supplemented with 10% bovine serum with apparent dissociation constant (K_D_) of 0.02 ± 0.01 units/mL (*n* = 4; error bars are SEM). Binding affinity is not statistically different compared to sensor performance in buffer (*p*-value ~0.3). (**B**) Biosensor binding of CD AABs compared to binding of other soluble proteins used as off-target biomarkers: GAPDH IgG (1 U/mL, a structurally similar antibody) and Myc/Max (200 nM, transcription factor complex with non-specific DNA-association properties that could have caused false-positive signals). Asterisks indicate statistically significant (*** *p*-value < 0.001 and ** *p*-value < 0.01) differences in the listed pairwise comparisons, where statistical analysis was performed using a 2-tailed Student’s *t*-test with unequal variance.

Upon testing our CD AAB biosensors with varying concentrations of CD AABs, we found that the biosensors detected the AABs with a limit of detection of ~10^−2^ × units/mL, with an ~80-fold dynamic range of detection (common for this class of biosensors [20]). Following equilibration, the biosensors rapidly and reproducibly bound CD AABs diluted in binding buffer (Figure 2B, data available in Supplemental Table S2). A 63% signal decrease was observed between the lowest (0.01 U/mL) to highest (10 U/mL) concentrations tested, with an apparent K_D_ of 0.09 ± 0.03 units/mL (*R*^2^ = 0.851; Sy.x = 0.092). No significant current change was observed when biosensors were challenged with binding buffer alone (Supplemental Figure S1). When biosensors were similarly equilibrated and tested for their ability to bind AABs in binding buffer supplemented with 10% bovine serum, we observed a small, statistically insignificant (*p*-value ~0.3) change in apparent affinity to 0.02 ± 0.01 units/mL (*R*^2^ = 0.879; Sy.x of 0.0694) (Figure 3A, data available in Supplemental Table S3). The biosensor was additionally tested with off-target proteins; specifically, anti-GAPDH antibodies and the Myc/Max transcription factor complex. The Myc/Max transcription factor complex was chosen because it has non-specific DNA-association properties, allowing us to test a non-specific DNA-association biosensor response. Upon challenging the biosensor with these off-target proteins, a significantly reduced current response was produced with both the anti-GAPDH antibody (1 U/mL) and Myc/Max transcription factor complex (200 nM), compared to CD AAB binding (*p*-value < 0.001 and *p*-value < 0.01, respectively; one outlier was excluded from analysis via Grubb’s test and Chauvenet’s criterion) (Figure 3B, data available in Supplemental Table S4).

## 4. Discussion

The E-DNA biosensor presented here showed sensitive and specific detection of CD AABs. We found the neoepitope employed here, naturally found in the crosslinking of tTG and undigested gliadin fragments, to be capable of being bound by CD IgA AABs in this E-DNA biosensor system. Previously, this neoepitope was found to be detected by CD IgG AABs in an ELISA [8]. Our biosensor presented here meets sensitivity sufficient for the clinically relevant range (~6 units/mL); therefore, further optimization of this system may make it feasible for routine CD serological tests and would help with overcoming the need for diagnostic biopsies [7]. The biosensor also showed high specificity, because challenges with off-target antibodies or protein complexes with non-specific DNA-binding properties (Figure 3B) or testing in binding buffer alone (Supplementary Figure S1) resulted in significantly reduced current changes. However, relatively large error was observed (Figure 2B and Figure 3A), which we believe was due to instrumentation limitations (noise and decreased resolution at the μA level) and baseline drift of the E-DNA system. Drift has been reduced by others by collecting dual measurements at both an active and inactive frequency (known as the kinetic differential measurement) [21]. We believe that future work incorporating such drift reduction techniques and utilizing instrumentation with increased resolution would significantly reduce the observed error.

Although the biosensor displayed well-behaved and sensitive binding in physiologically relevant buffer conditions (our PBS-based binding buffer is a common isotonic buffer for protein and cell studies; the added MgCl_2_ aids in proper aptamer folding and Tween-20 aids in preventing non-specific protein aggregation), when initial biosensor assessments were performed in undiluted, whole blood serum, no measurable biosensor response was detected, leading to the assessment in buffer supplemented with 10% bovine serum. When biosensors were tested in this binding buffer supplemented with 10% bovine serum (Figure 3A), signal quality was low, with considerable noise, limiting the utility of the current design in medical diagnostics. This reduced signal quality in complex media has been previously observed [11,19], and future efforts should be aimed at increasing signal quality by building on a number of strategies, including modifying the voltametric interrogation or designing physical barriers to isolate the working surface from the complex media, which has been shown to increase the reliability in complex media [19]. Future work will focus on creating a CD AAB biosensor that shows increased sensitivity to detection in serum. Others have shown that increased flexibility of DNA probes can improve the signal-to-noise ratio [22]. Additionally, incorporating a polyethylene glycol spacer between the anchor strand and the gold surface has shown positive results in protein-based electrochemical biosensors [23]. Such improvements could allow us to expand the use of similar scaffold biosensors for the detection of diseases with large biomarkers that would otherwise overload conventional E-DNA biosensors.

## 5. Conclusions

This work supports the growing field of E-DNA biosensors being used as versatile and broadly applicable diagnostic tools for a range of biomarkers. This biosensor displays robust response to CD AABs, at a concentration below the clinically relevant range, and has high specificity with minimal signal change when challenged with off-targets. The main limitation of the current work is the poor performance in binding buffer supplemented with serum, although recent publications have presented strategies to improve the performance of these types of biosensors, and could be incorporated into future, more optimized designs. Thus, the general approach employed here, and with other biosensors, presents a platform of readily generating biosensors for suitable diagnostic purposes, especially high molecular weight targets. Ultimately, such E-DNA biosensors may find great utility in a range of diagnostics.

## Data Availability

The data presented in this study are available in Supplementary Tables S1–S4.

## Supplementary Materials

The following are available online at https://www.mdpi.com/article/10.3390/s21082671/s1, Table S1: Voltametric data; Table S2: Normalized voltametric peak height titration data; Figure S1: Buffer control data; Table S3: Normalized voltametric peak height titration in serum data; and Table S4: Voltametric data for off-target analysis.
