# Supplementary material for: Electrochemical DNA Biosensor That Detects Early Celiac Disease Autoantibodies
Source: Sensors (Basel). 2021 Apr 10;21(8):2671. doi: 10.3390/s21082671 (PMC8070315; doi:10.3390/s21082671)
Supplement: Supplementary file 1 [file sensors-21-02671-s001.pdf]

Supplementary Data for "Electrochemical DNA biosensor that detects early Celiac Disease autoantibodies".

Anna B.N. Nguyen, Marcos Maldonado, Dylan Poch, Tyler Sodia, Andrew Smith, Teisha J. Rowland, and Andrew J. Bonham

Below is the data used to generate the figures in the article text.

Table S1. voltametric data for **Figure 2A**.

Potential (V)	DNA	PNA	AABs
-0.499868	1.52E-06	9.89E-07	1.3E-06
-0.499351	1.94E-06	1.29E-06	1.57E-06
-0.498963	1.93E-06	1.32E-06	1.55E-06
-0.498187	1.84E-06	1.27E-06	1.58E-06
-0.497864	1.83E-06	1.29E-06	1.54E-06
-0.497476	1.78E-06	1.19E-06	1.43E-06
-0.496894	1.7E-06	1.15E-06	1.4E-06
-0.496247	1.69E-06	1.23E-06	1.57E-06
-0.49573	1.72E-06	1.16E-06	1.49E-06
-0.495471	1.71E-06	1.13E-06	1.5E-06
-0.494824	1.67E-06	1.12E-06	1.4E-06
-0.494437	1.65E-06	1.23E-06	1.35E-06
-0.493919	1.6E-06	1.08E-06	1.34E-06
-0.493272	1.61E-06	1.16E-06	1.38E-06
-0.492884	1.6E-06	1.15E-06	1.34E-06
-0.492367	1.61E-06	1.16E-06	1.34E-06
-0.49185	1.55E-06	1.1E-06	1.35E-06
-0.491268	1.56E-06	1.16E-06	1.34E-06
-0.490815	1.54E-06	1.14E-06	1.41E-06
-0.490363	1.55E-06	1.12E-06	1.36E-06
-0.489781	1.52E-06	1.1E-06	1.31E-06
-0.489263	1.47E-06	1.04E-06	1.29E-06
-0.488746	1.48E-06	1.06E-06	1.26E-06
-0.488293	1.54E-06	1.12E-06	1.23E-06
-0.48797	1.49E-06	1.08E-06	1.28E-06
-0.487453	1.4E-06	1.01E-06	1.3E-06
-0.486871	1.45E-06	1.01E-06	1.18E-06
-0.486418	1.44E-06	1.08E-06	1.23E-06
-0.485965	1.43E-06	1.1E-06	1.23E-06
-0.485383	1.39E-06	1.01E-06	1.25E-06
-0.484995	1.43E-06	1.02E-06	1.25E-06
-0.484413	1.38E-06	1.06E-06	1.27E-06
-0.483767	1.42E-06	1.02E-06	1.27E-06

-0.483508	1.42E-06	1.03E-06	1.25E-06
-0.482797	1.33E-06	1.04E-06	1.21E-06
-0.482279	1.38E-06	1.02E-06	1.17E-06
-0.481827	1.42E-06	1.04E-06	1.17E-06
-0.481439	1.36E-06	9.58E-07	1.24E-06
-0.480857	1.37E-06	1.01E-06	1.23E-06
-0.480469	1.36E-06	1.05E-06	1.21E-06
-0.479887	1.33E-06	9.68E-07	1.2E-06
-0.479305	1.33E-06	1.03E-06	1.15E-06
-0.478852	1.32E-06	9.73E-07	1.19E-06
-0.478464	1.31E-06	1.03E-06	1.16E-06
-0.477882	1.32E-06	9.62E-07	1.08E-06
-0.47743	1.35E-06	9.33E-07	1.14E-06
-0.476977	1.31E-06	9.29E-07	1.15E-06
-0.476136	1.31E-06	9.52E-07	1.1E-06
-0.475813	1.28E-06	9.39E-07	1.17E-06
-0.475425	1.26E-06	1.01E-06	1.17E-06
-0.474908	1.32E-06	9.44E-07	1.1E-06
-0.474261	1.28E-06	8.6E-07	1.09E-06
-0.473679	1.23E-06	1.05E-06	1.17E-06
-0.473291	1.27E-06	9.42E-07	1.17E-06
-0.472709	1.31E-06	8.57E-07	1.1E-06
-0.472386	1.25E-06	9.65E-07	1.06E-06
-0.471739	1.28E-06	9.83E-07	1.2E-06
-0.471222	1.31E-06	8.39E-07	1.15E-06
-0.470898	1.19E-06	9.28E-07	1.03E-06
-0.470381	1.21E-06	9.25E-07	1.09E-06
-0.469928	1.23E-06	9.12E-07	1.03E-06
-0.469346	1.21E-06	9.89E-07	1.05E-06
-0.469023	1.19E-06	9.39E-07	1.02E-06
-0.468441	1.21E-06	9.13E-07	1.07E-06
-0.467924	1.16E-06	9.47E-07	1.1E-06
-0.467471	1.17E-06	9.73E-07	1.06E-06
-0.466824	1.13E-06	8.49E-07	1.02E-06
-0.466242	1.12E-06	9.09E-07	1.08E-06
-0.465855	1.17E-06	9.26E-07	1.06E-06
-0.465467	1.24E-06	9.36E-07	1.05E-06
-0.464949	1.16E-06	9.44E-07	1.03E-06
-0.464302	1.2E-06	9.52E-07	1.08E-06
-0.463915	1.19E-06	9.1E-07	1.02E-06
-0.463333	1.16E-06	8.59E-07	1.01E-06
-0.462945	1.2E-06	9.36E-07	1.05E-06
-0.462233	1.16E-06	8.91E-07	1.11E-06

-0.461781	1.14E-06	8.39E-07	1E-06
-0.461393	1.14E-06	8.89E-07	1.02E-06
-0.460811	1.14E-06	8.71E-07	1.04E-06
-0.460358	1.16E-06	9.09E-07	1.08E-06
-0.45997	1.07E-06	8.43E-07	1.1E-06
-0.459323	1.07E-06	9.13E-07	1.02E-06
-0.458806	1.08E-06	9.05E-07	1.09E-06
-0.458483	1.12E-06	8.68E-07	1.06E-06
-0.457836	1.09E-06	8.7E-07	1.04E-06
-0.457448	1.13E-06	8.02E-07	1.03E-06
-0.456801	1.14E-06	8.41E-07	1.03E-06
-0.456413	1.12E-06	7.59E-07	1.06E-06
-0.455831	1.12E-06	8.81E-07	9.55E-07
-0.455379	1.12E-06	9.07E-07	9.71E-07
-0.454861	1.13E-06	9.12E-07	1.02E-06
-0.454409	1.11E-06	8.73E-07	9.05E-07
-0.453762	1.06E-06	8.05E-07	1E-06
-0.45318	1.1E-06	8.05E-07	1.04E-06
-0.452857	1.13E-06	7.99E-07	9.67E-07
-0.452275	1.1E-06	7.97E-07	9.57E-07
-0.451693	1.12E-06	8.54E-07	1.05E-06
-0.451176	1.13E-06	8.1E-07	1E-06
-0.450852	1.09E-06	8.1E-07	9.62E-07
-0.450335	1.07E-06	8.97E-07	9.92E-07
-0.449753	1.06E-06	8.39E-07	9.92E-07
-0.449494	1.04E-06	8.46E-07	9.36E-07
-0.448783	1.06E-06	8.01E-07	1.02E-06
-0.448266	9.94E-07	8.84E-07	1E-06
-0.447813	1.07E-06	8.31E-07	1.04E-06
-0.447425	1.05E-06	8.41E-07	1.01E-06
-0.446778	1.1E-06	8.68E-07	9.73E-07
-0.446455	1.06E-06	8.25E-07	1E-06
-0.445808	9.99E-07	8.6E-07	9.34E-07
-0.445356	1.02E-06	8.1E-07	9.31E-07
-0.444645	1.04E-06	8.92E-07	9.36E-07
-0.444127	1.07E-06	8.55E-07	9.81E-07
-0.443739	1.05E-06	8.51E-07	9.6E-07
-0.443351	1.06E-06	8.23E-07	9.31E-07
-0.442963	1.01E-06	7.57E-07	9.94E-07
-0.442316	1.04E-06	8.1E-07	9.46E-07
-0.441864	1.07E-06	8.1E-07	9.87E-07
-0.441346	1.03E-06	8.2E-07	9.99E-07
-0.440764	1.04E-06	8.05E-07	1.03E-06

-0.440247	1.03E-06	8.55E-07	9.68E-07
-0.439859	1.09E-06	7.81E-07	9.81E-07
-0.439407	1.03E-06	8.22E-07	9.76E-07
-0.438889	1.05E-06	8.15E-07	9.04E-07
-0.438372	9.68E-07	8.63E-07	9.94E-07
-0.43779	1.08E-06	8.02E-07	9.28E-07
-0.437208	1.07E-06	8.39E-07	8.71E-07
-0.436691	1.04E-06	8.52E-07	9.68E-07
-0.436238	1.01E-06	8.62E-07	9.81E-07
-0.435785	9.64E-07	7.88E-07	9.1E-07
-0.435333	1.03E-06	8.81E-07	9.91E-07
-0.434945	1.06E-06	8.25E-07	9.65E-07
-0.434363	1.02E-06	8.54E-07	9.55E-07
-0.43391	1.03E-06	8.05E-07	9.89E-07
-0.433393	1.02E-06	7.65E-07	9.92E-07
-0.432681	1.03E-06	7.99E-07	9.25E-07
-0.432293	1.01E-06	9.23E-07	9.05E-07
-0.431841	9.7E-07	8.8E-07	8.78E-07
-0.431323	1.03E-06	9.18E-07	9.83E-07
-0.431	1.02E-06	8.52E-07	9.78E-07
-0.430483	9.41E-07	8.04E-07	9.62E-07
-0.429771	9.77E-07	8.91E-07	9.92E-07
-0.429383	1.02E-06	8.38E-07	9.55E-07
-0.428737	9.91E-07	8.78E-07	9.23E-07
-0.428284	1.03E-06	8.49E-07	9.09E-07
-0.427831	9.82E-07	8.07E-07	9.09E-07
-0.427314	1.02E-06	8.51E-07	9.17E-07
-0.426861	9.72E-07	8.42E-07	9.75E-07
-0.426344	9.81E-07	8.88E-07	9.49E-07
-0.426021	9.52E-07	8.12E-07	9.84E-07
-0.425374	1.06E-06	8.25E-07	9.68E-07
-0.424728	9.64E-07	8.38E-07	9.89E-07
-0.424275	9.36E-07	8.36E-07	9.54E-07
-0.423887	9.67E-07	8.92E-07	9.78E-07
-0.423499	1.02E-06	8.15E-07	9.41E-07
-0.422917	1.05E-06	9.05E-07	9.39E-07
-0.422335	1.02E-06	8.31E-07	9.73E-07
-0.421947	9.73E-07	8.39E-07	8.88E-07
-0.421365	1.06E-06	8.41E-07	9.5E-07
-0.420718	9.9E-07	8.81E-07	9.47E-07
-0.420524	1.09E-06	9.07E-07	9.79E-07
-0.419942	1.01E-06	9.58E-07	9.76E-07
-0.41949	1.08E-06	9.04E-07	9.73E-07

-0.418843	1.01E-06	8.91E-07	9.36E-07
-0.418455	1.02E-06	9.36E-07	9.62E-07
-0.417873	9.86E-07	8.97E-07	9.68E-07
-0.417291	1.01E-06	9.67E-07	9.55E-07
-0.416903	1.04E-06	1E-06	9.52E-07
-0.416515	1.06E-06	9.18E-07	1.01E-06
-0.415868	1.06E-06	9.04E-07	9.52E-07
-0.41561	9.95E-07	9.52E-07	9.73E-07
-0.415028	1.05E-06	9.31E-07	1.06E-06
-0.414316	1.04E-06	9.1E-07	9.47E-07
-0.413929	9.77E-07	9.78E-07	9.78E-07
-0.413411	1E-06	8.83E-07	9.84E-07
-0.412894	1.02E-06	9.26E-07	9.52E-07
-0.412635	1.09E-06	9.04E-07	1.02E-06
-0.41173	1.09E-06	9.34E-07	1E-06
-0.411407	1.06E-06	9.29E-07	1.04E-06
-0.410889	1.03E-06	9.87E-07	1.06E-06
-0.410307	1.12E-06	1.06E-06	1.12E-06
-0.409919	1.07E-06	9.87E-07	1.09E-06
-0.409337	1.08E-06	9.97E-07	1.08E-06
-0.408949	1.08E-06	9.62E-07	1.01E-06
-0.408367	1.04E-06	1.04E-06	1.07E-06
-0.407915	1.13E-06	9.99E-07	1.06E-06
-0.407462	1.14E-06	1.07E-06	1.13E-06
-0.40688	1.14E-06	9.89E-07	1.09E-06
-0.406492	1.1E-06	1.06E-06	1.06E-06
-0.405975	1.14E-06	1.08E-06	1.2E-06
-0.405651	1.09E-06	1.11E-06	1.04E-06
-0.405005	1.13E-06	1.08E-06	1.06E-06
-0.404423	1.1E-06	1.06E-06	1.1E-06
-0.40397	1.1E-06	1.07E-06	1.02E-06
-0.403517	1.14E-06	1.14E-06	1.17E-06
-0.402935	1.16E-06	1.08E-06	1.17E-06
-0.402418	1.14E-06	1.16E-06	1.11E-06
-0.402095	1.06E-06	1.11E-06	1.19E-06
-0.401383	1.16E-06	1.13E-06	1.17E-06
-0.400931	1.18E-06	1.16E-06	1.15E-06
-0.400284	1.15E-06	1.17E-06	1.15E-06
-0.400025	1.15E-06	1.17E-06	1.23E-06
-0.399444	1.15E-06	1.15E-06	1.25E-06
-0.398991	1.22E-06	1.19E-06	1.23E-06
-0.398603	1.2E-06	1.21E-06	1.19E-06
-0.397956	1.2E-06	1.26E-06	1.23E-06

-0.397051	1.22E-06	1.16E-06	1.21E-06
-0.396922	1.21E-06	1.24E-06	1.38E-06
-0.396534	1.24E-06	1.34E-06	1.23E-06
-0.395822	1.26E-06	1.23E-06	1.34E-06
-0.395434	1.26E-06	1.34E-06	1.27E-06
-0.394723	1.22E-06	1.3E-06	1.31E-06
-0.394206	1.3E-06	1.27E-06	1.49E-06
-0.393882	1.29E-06	1.34E-06	1.3E-06
-0.3933	1.33E-06	1.36E-06	1.3E-06
-0.392912	1.3E-06	1.39E-06	1.46E-06
-0.392395	1.27E-06	1.44E-06	1.36E-06
-0.391813	1.29E-06	1.47E-06	1.48E-06
-0.391425	1.38E-06	1.5E-06	1.45E-06
-0.390843	1.46E-06	1.42E-06	1.43E-06
-0.390326	1.44E-06	1.53E-06	1.57E-06
-0.389873	1.4E-06	1.5E-06	1.47E-06
-0.38955	1.35E-06	1.55E-06	1.6E-06
-0.388903	1.42E-06	1.58E-06	1.47E-06
-0.388256	1.5E-06	1.54E-06	1.49E-06
-0.387739	1.52E-06	1.63E-06	1.67E-06
-0.387481	1.48E-06	1.55E-06	1.51E-06
-0.386963	1.54E-06	1.57E-06	1.62E-06
-0.386511	1.54E-06	1.54E-06	1.54E-06
-0.385799	1.56E-06	1.69E-06	1.54E-06
-0.385476	1.55E-06	1.69E-06	1.72E-06
-0.384829	1.52E-06	1.79E-06	1.54E-06
-0.384312	1.65E-06	1.8E-06	1.79E-06
-0.383795	1.63E-06	1.8E-06	1.61E-06
-0.383342	1.67E-06	1.83E-06	1.58E-06
-0.382695	1.65E-06	1.88E-06	1.95E-06
-0.382372	1.69E-06	1.81E-06	1.66E-06
-0.38179	1.68E-06	1.89E-06	1.88E-06
-0.381467	1.72E-06	1.94E-06	1.8E-06
-0.38082	1.77E-06	1.92E-06	1.77E-06
-0.380432	1.79E-06	1.97E-06	1.9E-06
-0.379785	1.76E-06	2.03E-06	1.84E-06
-0.379268	1.81E-06	2E-06	2.01E-06
-0.378945	1.85E-06	2.13E-06	1.88E-06
-0.378298	1.83E-06	2.1E-06	1.88E-06
-0.377781	1.97E-06	2.17E-06	2.14E-06
-0.377199	1.88E-06	2.16E-06	1.94E-06
-0.376746	1.87E-06	2.17E-06	2.16E-06
-0.376229	1.98E-06	2.31E-06	1.9E-06

-0.375841	1.99E-06	2.22E-06	2.06E-06
-0.375259	2.06E-06	2.26E-06	2.2E-06
-0.374741	2.03E-06	2.3E-06	2.07E-06
-0.374353	2.06E-06	2.32E-06	2.3E-06
-0.373901	2.13E-06	2.39E-06	2.08E-06
-0.373384	2.15E-06	2.39E-06	2.14E-06
-0.372931	2.16E-06	2.43E-06	2.28E-06
-0.372284	2.21E-06	2.51E-06	2.25E-06
-0.371831	2.24E-06	2.44E-06	2.33E-06
-0.371314	2.25E-06	2.59E-06	2.23E-06
-0.370991	2.25E-06	2.57E-06	2.29E-06
-0.37028	2.35E-06	2.66E-06	2.45E-06
-0.369892	2.38E-06	2.61E-06	2.3E-06
-0.369439	2.44E-06	2.71E-06	2.4E-06
-0.368792	2.38E-06	2.74E-06	2.4E-06
-0.368081	2.47E-06	2.77E-06	2.41E-06
-0.367952	2.53E-06	2.8E-06	2.45E-06
-0.36737	2.57E-06	2.83E-06	2.52E-06
-0.366852	2.56E-06	2.92E-06	2.52E-06
-0.366206	2.53E-06	2.86E-06	2.42E-06
-0.365753	2.71E-06	2.91E-06	2.54E-06
-0.3653	2.73E-06	2.99E-06	2.51E-06
-0.364783	2.74E-06	3.03E-06	2.6E-06
-0.364201	2.81E-06	3.04E-06	2.57E-06
-0.363942	2.82E-06	3.13E-06	2.75E-06
-0.36336	2.94E-06	3.18E-06	2.78E-06
-0.362843	2.93E-06	3.16E-06	2.78E-06
-0.362261	2.89E-06	3.2E-06	2.77E-06
-0.361808	2.94E-06	3.3E-06	2.78E-06
-0.361226	2.93E-06	3.21E-06	2.85E-06
-0.360774	3.04E-06	3.33E-06	2.87E-06
-0.360321	3.08E-06	3.45E-06	2.87E-06
-0.359869	3.14E-06	3.37E-06	3.03E-06
-0.359222	3.19E-06	3.41E-06	3E-06
-0.358704	3.2E-06	3.45E-06	3.01E-06
-0.358317	3.3E-06	3.5E-06	3.03E-06
-0.357864	3.28E-06	3.61E-06	3.17E-06
-0.357217	3.34E-06	3.61E-06	3.16E-06
-0.356894	3.45E-06	3.62E-06	3.15E-06
-0.356247	3.4E-06	3.66E-06	3.24E-06
-0.355795	3.48E-06	3.76E-06	3.27E-06
-0.355213	3.46E-06	3.84E-06	3.18E-06
-0.35476	3.51E-06	3.76E-06	3.33E-06

-0.354307	3.56E-06	3.88E-06	3.3E-06
-0.35379	3.65E-06	3.92E-06	3.37E-06
-0.353208	3.69E-06	3.86E-06	3.35E-06
-0.352949	3.79E-06	3.87E-06	3.46E-06
-0.352173	3.8E-06	3.99E-06	3.49E-06
-0.351785	3.83E-06	4.05E-06	3.56E-06
-0.351203	3.94E-06	4.03E-06	3.54E-06
-0.350686	3.96E-06	4.1E-06	3.6E-06
-0.350233	3.94E-06	4.12E-06	3.58E-06
-0.349845	4.02E-06	4.21E-06	3.62E-06
-0.349199	4.06E-06	4.18E-06	3.65E-06
-0.348811	4.14E-06	4.22E-06	3.71E-06
-0.348229	4.15E-06	4.26E-06	3.73E-06
-0.347776	4.11E-06	4.35E-06	3.69E-06
-0.347194	4.19E-06	4.3E-06	3.85E-06
-0.346741	4.28E-06	4.37E-06	3.88E-06
-0.346289	4.22E-06	4.39E-06	3.95E-06
-0.345707	4.35E-06	4.42E-06	3.89E-06
-0.34506	4.38E-06	4.47E-06	3.99E-06
-0.344672	4.46E-06	4.51E-06	4.03E-06
-0.34409	4.46E-06	4.47E-06	4.08E-06
-0.343702	4.51E-06	4.58E-06	4E-06
-0.342991	4.54E-06	4.54E-06	4.04E-06
-0.342732	4.55E-06	4.62E-06	4.05E-06
-0.34215	4.61E-06	4.61E-06	4.09E-06
-0.341568	4.69E-06	4.71E-06	4.1E-06
-0.34118	4.64E-06	4.61E-06	4.16E-06
-0.340469	4.74E-06	4.73E-06	4.16E-06
-0.34021	4.86E-06	4.75E-06	4.16E-06
-0.339564	4.84E-06	4.76E-06	4.29E-06
-0.33924	4.79E-06	4.76E-06	4.24E-06
-0.338723	4.89E-06	4.77E-06	4.28E-06
-0.338206	4.98E-06	4.79E-06	4.31E-06
-0.337753	4.91E-06	4.86E-06	4.27E-06
-0.337236	4.93E-06	4.94E-06	4.37E-06
-0.336524	5.1E-06	4.98E-06	4.31E-06
-0.336136	5.13E-06	4.96E-06	4.4E-06
-0.335748	5.09E-06	4.97E-06	4.5E-06
-0.335231	5.16E-06	5.01E-06	4.42E-06
-0.334714	5.18E-06	5.02E-06	4.46E-06
-0.334132	5.24E-06	4.96E-06	4.56E-06
-0.333744	5.29E-06	5E-06	4.57E-06
-0.333291	5.31E-06	5.05E-06	4.58E-06

-0.332709	5.27E-06	5.1E-06	4.56E-06
-0.332257	5.31E-06	5.09E-06	4.66E-06
-0.331675	5.42E-06	5.1E-06	4.68E-06
-0.331093	5.4E-06	5.11E-06	4.6E-06
-0.33064	5.41E-06	5.03E-06	4.59E-06
-0.330123	5.41E-06	5.22E-06	4.59E-06
-0.32967	5.5E-06	5.13E-06	4.61E-06
-0.329153	5.53E-06	5.13E-06	4.66E-06
-0.328765	5.55E-06	5.16E-06	4.71E-06
-0.328118	5.52E-06	5.1E-06	4.7E-06
-0.32773	5.58E-06	5.23E-06	4.76E-06
-0.327277	5.55E-06	5.2E-06	4.68E-06
-0.326631	5.64E-06	5.24E-06	4.82E-06
-0.326178	5.65E-06	5.3E-06	4.76E-06
-0.325661	5.71E-06	5.25E-06	4.78E-06
-0.325079	5.68E-06	5.18E-06	4.86E-06
-0.324691	5.68E-06	5.22E-06	4.84E-06
-0.324238	5.71E-06	5.29E-06	4.8E-06
-0.323591	5.71E-06	5.18E-06	4.75E-06
-0.323268	5.74E-06	5.17E-06	4.86E-06
-0.322815	5.78E-06	5.21E-06	4.79E-06
-0.322104	5.8E-06	5.28E-06	4.75E-06
-0.321652	5.82E-06	5.3E-06	4.84E-06
-0.321199	5.81E-06	5.25E-06	4.92E-06
-0.320746	5.79E-06	5.14E-06	4.78E-06
-0.320229	5.82E-06	5.23E-06	4.92E-06
-0.319711	5.86E-06	5.23E-06	4.81E-06
-0.319259	5.94E-06	5.22E-06	4.81E-06
-0.318677	5.82E-06	5.22E-06	4.79E-06
-0.318289	5.89E-06	5.2E-06	4.78E-06
-0.317707	5.85E-06	5.15E-06	4.87E-06
-0.317319	5.84E-06	5.09E-06	4.83E-06
-0.316931	5.88E-06	5.15E-06	4.86E-06
-0.316026	5.91E-06	5.26E-06	4.84E-06
-0.315638	5.84E-06	5.11E-06	4.81E-06
-0.31525	5.83E-06	5.1E-06	4.8E-06
-0.314732	5.86E-06	5.12E-06	4.77E-06
-0.31428	5.89E-06	5.1E-06	4.94E-06
-0.313762	5.91E-06	5.11E-06	4.79E-06
-0.31318	5.84E-06	5.11E-06	4.81E-06
-0.312857	5.93E-06	5.1E-06	4.8E-06
-0.312081	5.93E-06	5.11E-06	4.74E-06
-0.311887	5.9E-06	5.1E-06	4.79E-06

-0.311111	5.88E-06	5.03E-06	4.76E-06
-0.310788	5.86E-06	5E-06	4.82E-06
-0.310141	5.81E-06	5.08E-06	4.85E-06
-0.309753	5.83E-06	4.97E-06	4.8E-06
-0.3093	5.82E-06	4.86E-06	4.68E-06
-0.308654	5.81E-06	4.95E-06	4.71E-06
-0.308266	5.79E-06	4.87E-06	4.65E-06
-0.307813	5.75E-06	4.88E-06	4.71E-06
-0.307231	5.77E-06	4.87E-06	4.62E-06
-0.306908	5.83E-06	4.77E-06	4.67E-06
-0.30652	5.75E-06	4.86E-06	4.62E-06
-0.305744	5.72E-06	4.84E-06	4.58E-06
-0.305162	5.74E-06	4.76E-06	4.53E-06
-0.304903	5.72E-06	4.76E-06	4.58E-06
-0.304127	5.67E-06	4.67E-06	4.56E-06
-0.303739	5.72E-06	4.66E-06	4.61E-06
-0.303287	5.66E-06	4.57E-06	4.53E-06
-0.302899	5.62E-06	4.63E-06	4.5E-06
-0.302317	5.62E-06	4.6E-06	4.47E-06
-0.301799	5.64E-06	4.57E-06	4.5E-06
-0.301217	5.61E-06	4.54E-06	4.47E-06
-0.300894	5.53E-06	4.53E-06	4.41E-06
-0.300312	5.58E-06	4.48E-06	4.39E-06
-0.299795	5.6E-06	4.41E-06	4.28E-06
-0.299342	5.48E-06	4.35E-06	4.41E-06
-0.298889	5.43E-06	4.46E-06	4.3E-06
-0.298243	5.43E-06	4.34E-06	4.27E-06
-0.29779	5.42E-06	4.28E-06	4.27E-06
-0.297337	5.4E-06	4.2E-06	4.12E-06
-0.296885	5.34E-06	4.29E-06	4.22E-06
-0.296238	5.37E-06	4.2E-06	4.19E-06
-0.295785	5.29E-06	4.19E-06	4.2E-06
-0.295139	5.26E-06	4.19E-06	4.14E-06
-0.294815	5.27E-06	4.17E-06	4.14E-06
-0.294169	5.22E-06	4.08E-06	4.16E-06
-0.293652	5.19E-06	4.06E-06	4E-06
-0.293199	5.23E-06	4.13E-06	4.01E-06
-0.292746	5.14E-06	3.92E-06	3.99E-06
-0.292229	5.11E-06	3.92E-06	4.01E-06
-0.291647	5.13E-06	3.92E-06	3.98E-06
-0.29113	5.04E-06	3.86E-06	3.92E-06
-0.290871	4.98E-06	3.84E-06	3.95E-06
-0.29016	4.9E-06	3.9E-06	3.83E-06

-0.289642	4.94E-06	3.76E-06	3.86E-06
-0.289254	4.83E-06	3.66E-06	3.75E-06
-0.288672	4.85E-06	3.74E-06	3.74E-06
-0.288284	4.8E-06	3.65E-06	3.76E-06
-0.287702	4.8E-06	3.67E-06	3.61E-06
-0.287056	4.74E-06	3.58E-06	3.62E-06
-0.286862	4.68E-06	3.55E-06	3.69E-06
-0.286021	4.61E-06	3.49E-06	3.6E-06
-0.285698	4.6E-06	3.49E-06	3.63E-06
-0.28531	4.61E-06	3.47E-06	3.52E-06
-0.284792	4.52E-06	3.43E-06	3.51E-06
-0.284275	4.46E-06	3.5E-06	3.5E-06
-0.283629	4.41E-06	3.46E-06	3.45E-06
-0.283176	4.39E-06	3.21E-06	3.42E-06
-0.282788	4.36E-06	3.24E-06	3.43E-06
-0.282206	4.31E-06	3.19E-06	3.33E-06
-0.281753	4.31E-06	3.14E-06	3.33E-06
-0.281365	4.2E-06	3.16E-06	3.3E-06
-0.280718	4.25E-06	3.08E-06	3.23E-06
-0.280266	4.16E-06	3.08E-06	3.18E-06
-0.279684	4.14E-06	3.07E-06	3.12E-06
-0.279167	4.08E-06	3.04E-06	3.18E-06
-0.278714	4.09E-06	3.01E-06	3.14E-06
-0.278132	4.03E-06	2.91E-06	3.06E-06
-0.277679	4.03E-06	2.88E-06	2.92E-06
-0.277162	3.93E-06	2.9E-06	3.05E-06
-0.276645	3.86E-06	2.87E-06	3.09E-06
-0.276321	3.91E-06	2.84E-06	2.96E-06
-0.275804	3.83E-06	2.79E-06	2.84E-06
-0.275416	3.77E-06	2.69E-06	2.85E-06
-0.27464	3.75E-06	2.63E-06	2.82E-06
-0.274317	3.7E-06	2.64E-06	2.85E-06
-0.273799	3.66E-06	2.67E-06	2.74E-06
-0.273217	3.64E-06	2.62E-06	2.77E-06
-0.272829	3.52E-06	2.47E-06	2.79E-06
-0.272247	3.55E-06	2.51E-06	2.68E-06
-0.271859	3.41E-06	2.52E-06	2.66E-06
-0.271148	3.46E-06	2.42E-06	2.63E-06
-0.270695	3.37E-06	2.38E-06	2.57E-06
-0.270243	3.38E-06	2.43E-06	2.64E-06
-0.269984	3.28E-06	2.38E-06	2.55E-06
-0.269402	3.25E-06	2.39E-06	2.52E-06
-0.268691	3.22E-06	2.36E-06	2.48E-06

-0.268174	3.18E-06	2.28E-06	2.53E-06
-0.267656	3.16E-06	2.23E-06	2.46E-06
-0.267268	3.08E-06	2.25E-06	2.42E-06
-0.266815	3.1E-06	2.15E-06	2.4E-06
-0.265975	3.02E-06	2.09E-06	2.39E-06
-0.265781	3.04E-06	2.16E-06	2.33E-06
-0.265263	3.05E-06	2.07E-06	2.22E-06
-0.265005	2.96E-06	2.09E-06	2.28E-06
-0.2641	2.9E-06	2.06E-06	2.2E-06
-0.263841	2.94E-06	2.05E-06	2.25E-06
-0.26313	2.84E-06	1.97E-06	2.11E-06
-0.262742	2.78E-06	2E-06	2.2E-06
-0.262289	2.76E-06	1.91E-06	2.15E-06
-0.261707	2.71E-06	1.98E-06	2.1E-06
-0.261254	2.65E-06	1.88E-06	2.16E-06
-0.260737	2.61E-06	1.89E-06	2.1E-06
-0.26022	2.58E-06	1.91E-06	2.03E-06
-0.259702	2.59E-06	1.9E-06	2.01E-06
-0.25925	2.56E-06	1.83E-06	1.94E-06
-0.258668	2.45E-06	1.74E-06	2.02E-06
-0.258344	2.45E-06	1.8E-06	1.94E-06
-0.257762	2.48E-06	1.74E-06	1.97E-06
-0.257439	2.42E-06	1.73E-06	1.88E-06
-0.256793	2.42E-06	1.7E-06	1.92E-06
-0.25634	2.33E-06	1.71E-06	1.87E-06
-0.255887	2.31E-06	1.68E-06	1.79E-06
-0.255241	2.26E-06	1.68E-06	1.81E-06
-0.254723	2.19E-06	1.59E-06	1.87E-06
-0.2544	2.2E-06	1.66E-06	1.83E-06
-0.253818	2.2E-06	1.54E-06	1.73E-06
-0.253301	2.2E-06	1.53E-06	1.68E-06
-0.252719	2.07E-06	1.55E-06	1.64E-06
-0.252266	2.09E-06	1.54E-06	1.69E-06
-0.252072	2.17E-06	1.53E-06	1.67E-06
-0.251296	2.04E-06	1.55E-06	1.59E-06
-0.250714	1.97E-06	1.51E-06	1.6E-06
-0.250391	1.89E-06	1.43E-06	1.62E-06
-0.249809	1.97E-06	1.49E-06	1.61E-06
-0.249485	1.94E-06	1.38E-06	1.61E-06
-0.248839	1.88E-06	1.4E-06	1.62E-06
-0.248386	1.81E-06	1.37E-06	1.57E-06
-0.247869	1.84E-06	1.31E-06	1.61E-06
-0.247416	1.79E-06	1.41E-06	1.52E-06

-0.246899	1.81E-06	1.3E-06	1.47E-06
-0.246187	1.76E-06	1.37E-06	1.43E-06
-0.245864	1.68E-06	1.37E-06	1.42E-06
-0.245411	1.77E-06	1.34E-06	1.42E-06
-0.244894	1.67E-06	1.28E-06	1.49E-06
-0.244247	1.64E-06	1.26E-06	1.39E-06
-0.243601	1.66E-06	1.27E-06	1.45E-06
-0.243342	1.67E-06	1.16E-06	1.44E-06
-0.242954	1.55E-06	1.21E-06	1.39E-06
-0.242307	1.55E-06	1.25E-06	1.3E-06
-0.241984	1.59E-06	1.19E-06	1.36E-06
-0.241402	1.53E-06	1.17E-06	1.41E-06
-0.24082	1.59E-06	1.15E-06	1.34E-06
-0.240303	1.5E-06	1.19E-06	1.37E-06
-0.239786	1.46E-06	1.12E-06	1.27E-06
-0.239397	1.47E-06	1.14E-06	1.3E-06
-0.23888	1.47E-06	1.1E-06	1.2E-06
-0.238169	1.44E-06	1.04E-06	1.22E-06
-0.23791	1.4E-06	1.08E-06	1.25E-06
-0.237328	1.46E-06	1.12E-06	1.21E-06
-0.237005	1.4E-06	1.07E-06	1.21E-06
-0.236294	1.38E-06	1.07E-06	1.18E-06
-0.235712	1.3E-06	1.11E-06	1.18E-06
-0.235388	1.36E-06	1.03E-06	1.21E-06
-0.234871	1.35E-06	1E-06	1.23E-06
-0.234289	1.32E-06	1.03E-06	1.15E-06
-0.233966	1.21E-06	1.01E-06	1.12E-06
-0.233319	1.25E-06	9.78E-07	1.2E-06
-0.232802	1.23E-06	1.02E-06	1.18E-06
-0.232543	1.26E-06	1.05E-06	1.18E-06
-0.231961	1.21E-06	9.79E-07	1.11E-06
-0.231315	1.21E-06	1.01E-06	1.17E-06
-0.230862	1.17E-06	9.99E-07	1.14E-06
-0.23028	1.15E-06	9.46E-07	1.01E-06
-0.229698	1.08E-06	1.02E-06	1.05E-06
-0.229439	1.2E-06	8.78E-07	1.14E-06
-0.228728	1.19E-06	9.1E-07	1.06E-06
-0.228405	1.1E-06	8.89E-07	1.05E-06
-0.227693	1.12E-06	9.78E-07	1.03E-06
-0.227435	1.09E-06	1.01E-06	1.05E-06
-0.226659	1.08E-06	9.18E-07	1.06E-06
-0.2264	1.08E-06	8.97E-07	1.01E-06
-0.225689	1.03E-06	9.17E-07	1.02E-06

-0.225301	1.06E-06	9.25E-07	9.71E-07
-0.224719	1.08E-06	8.63E-07	1.04E-06
-0.224331	1.01E-06	8.88E-07	1.03E-06
-0.223878	1.06E-06	8.33E-07	9.62E-07
-0.223231	1.02E-06	9.23E-07	9.23E-07
-0.222714	9.41E-07	8.33E-07	9.47E-07
-0.222455	9.88E-07	8.75E-07	9.5E-07
-0.221744	9.29E-07	8.28E-07	9.31E-07
-0.221356	9.02E-07	8.01E-07	9.58E-07
-0.220839	9.45E-07	8.71E-07	9.99E-07
-0.220257	9.16E-07	8.97E-07	9.13E-07
-0.219998	9.56E-07	8.07E-07	1.05E-06
-0.219351	8.4E-07	8.67E-07	9.02E-07
-0.218834	9.27E-07	8.52E-07	8.71E-07
-0.218188	9.06E-07	7.78E-07	9.71E-07
-0.217864	8.98E-07	7.75E-07	9.02E-07
-0.217347	9.25E-07	8.42E-07	9.25E-07
-0.2167	8.83E-07	8.34E-07	8.86E-07
-0.216506	8.69E-07	7.75E-07	9.02E-07
-0.215795	9.29E-07	8.18E-07	9.44E-07
-0.215407	8.52E-07	8.23E-07	8.8E-07
-0.214825	9.19E-07	7.38E-07	9.63E-07
-0.214243	8.44E-07	8.68E-07	9.39E-07
-0.213919	9.05E-07	8.15E-07	8.76E-07
-0.213338	7.23E-07	8.07E-07	8.71E-07
-0.212885	7.68E-07	8.01E-07	9.73E-07
-0.212368	8.42E-07	7.57E-07	9.26E-07
-0.211915	8.35E-07	8.15E-07	8.51E-07
-0.211204	7.65E-07	7.64E-07	8.42E-07
-0.210945	7.58E-07	7.6E-07	8.63E-07
-0.210298	8.29E-07	7.23E-07	7.91E-07
-0.209781	7.4E-07	7.15E-07	8.42E-07
-0.209458	8E-07	7.7E-07	9.07E-07
-0.20894	7.38E-07	6.85E-07	8.59E-07
-0.208358	7.58E-07	7.44E-07	8.81E-07
-0.207776	7.87E-07	6.59E-07	8.15E-07
-0.207324	7.87E-07	7.57E-07	8.55E-07
-0.206871	7.73E-07	7.35E-07	8.18E-07
-0.206483	7.69E-07	7.62E-07	8.31E-07
-0.205966	7.32E-07	6.83E-07	7.36E-07
-0.205449	7.59E-07	7.09E-07	8.76E-07
-0.204931	7.38E-07	7.09E-07	8.07E-07
-0.204479	7.59E-07	6.93E-07	8.12E-07

-0.204091	6.97E-07	5.94E-07	8.07E-07
-0.203444	6.73E-07	6.89E-07	7.78E-07
-0.202797	6.59E-07	6.25E-07	7.76E-07
-0.202474	6.82E-07	6.77E-07	7.81E-07
-0.201957	7.02E-07	6.93E-07	7.52E-07
-0.20131	6.31E-07	6.44E-07	8.04E-07
-0.200857	6.92E-07	6.98E-07	7.22E-07
-0.200405	6.74E-07	6.69E-07	7.73E-07
-0.200081	6.74E-07	6.41E-07	7.56E-07
-0.19937	6.8E-07	6.77E-07	7.3E-07
-0.199047	6.65E-07	6.2E-07	7.73E-07
-0.198271	6.22E-07	5.61E-07	7.81E-07
-0.197883	7.01E-07	6.15E-07	7.83E-07
-0.197365	6.38E-07	5.73E-07	7.62E-07
-0.196783	6.89E-07	6.4E-07	6.49E-07
-0.196395	6.51E-07	7.6E-07	7.89E-07
-0.195943	6.26E-07	6.36E-07	8.23E-07
-0.195231	6.07E-07	6.49E-07	7.76E-07
-0.194908	6.46E-07	6.65E-07	7.44E-07
-0.194326	6.19E-07	6.23E-07	7.65E-07
-0.193938	6.64E-07	6.59E-07	7.38E-07
-0.193356	6.38E-07	5.83E-07	7.2E-07
-0.192774	6.52E-07	5.56E-07	7.02E-07
-0.192386	6.04E-07	6.46E-07	7.89E-07
-0.191804	6.1E-07	6.11E-07	6.81E-07
-0.191416	5.97E-07	5.69E-07	7.14E-07
-0.190769	6.64E-07	6.19E-07	7.88E-07
-0.190317	5.95E-07	6.85E-07	6.64E-07
-0.189864	5.41E-07	6.28E-07	6.75E-07
-0.189412	5.95E-07	5.83E-07	7.15E-07
-0.188635	6.08E-07	6.3E-07	7.64E-07
-0.188442	5.85E-07	6.6E-07	7.01E-07
-0.187924	5.97E-07	5.69E-07	7.88E-07
-0.187407	5.5E-07	6.07E-07	7.1E-07
-0.186954	5.68E-07	7.07E-07	6.44E-07
-0.186437	6.02E-07	5.41E-07	6.62E-07
-0.185984	5.59E-07	7.02E-07	6.3E-07
-0.185596	5.7E-07	6.01E-07	7.22E-07
-0.185014	6.42E-07	5.51E-07	7.25E-07
-0.184497	6.06E-07	5.8E-07	7.62E-07
-0.183915	5.68E-07	5.69E-07	7.09E-07
-0.183527	5.85E-07	5.48E-07	6.65E-07
-0.182945	5.39E-07	5.01E-07	6.64E-07

-0.182428	5.9E-07	5.09E-07	6.49E-07
-0.18191	5.63E-07	6.75E-07	7.51E-07
-0.181458	5.49E-07	6.07E-07	6.91E-07
-0.181005	5.1E-07	5.75E-07	6.6E-07
-0.180553	5.15E-07	6.23E-07	6.62E-07
-0.17997	5.1E-07	6.33E-07	6.23E-07
-0.179388	5.59E-07	6.51E-07	6.49E-07
-0.178936	5.5E-07	5.07E-07	6.33E-07
-0.178483	6.01E-07	5.62E-07	6.91E-07
-0.177901	5.39E-07	5.07E-07	5.77E-07
-0.177578	5.27E-07	5.4E-07	5.65E-07
-0.176867	5.88E-07	4.25E-07	6.72E-07
-0.176414	5.5E-07	5.57E-07	6.6E-07
-0.175896	5.14E-07	5.28E-07	5.59E-07
-0.175379	4.65E-07	5.4E-07	6.02E-07
-0.175121	5.64E-07	5.25E-07	7.18E-07
-0.17428	5.5E-07	5.19E-07	6.81E-07
-0.173892	5.17E-07	5.64E-07	5.54E-07
-0.17331	5.27E-07	5.09E-07	6.22E-07
-0.172857	5.09E-07	4.62E-07	6.43E-07
-0.172405	5.13E-07	5.64E-07	5.88E-07
-0.171952	5.37E-07	5.27E-07	5.54E-07
-0.171499	5.82E-07	5.59E-07	5.46E-07
-0.170982	5.49E-07	5.73E-07	6.31E-07
-0.170335	6.26E-07	5.15E-07	6.25E-07
-0.170012	5.14E-07	4.49E-07	6.06E-07
-0.169365	5.52E-07	5.43E-07	6.83E-07
-0.168913	4.59E-07	4.95E-07	6.25E-07
-0.168525	4.86E-07	4.98E-07	5.99E-07
-0.167878	5.34E-07	5.67E-07	5.56E-07
-0.167361	5.03E-07	5.44E-07	6.12E-07
-0.167038	6.02E-07	4.66E-07	6.04E-07
-0.166456	5.1E-07	4.32E-07	6.19E-07
-0.166003	5.43E-07	5.25E-07	5.75E-07
-0.165485	4.76E-07	5.38E-07	5.94E-07
-0.164839	5.44E-07	4.86E-07	4.64E-07
-0.164515	4.94E-07	4.4E-07	5.91E-07
-0.164063	4.79E-07	5.12E-07	5.96E-07
-0.16361	4.67E-07	5.56E-07	5.99E-07
-0.16277	4.99E-07	5.04E-07	5.36E-07
-0.16264	4.48E-07	4.45E-07	5.28E-07
-0.161994	5.31E-07	4.56E-07	6.23E-07
-0.161347	5.15E-07	5.2E-07	5.72E-07

-0.16083	4.88E-07	5.27E-07	5.88E-07
-0.160377	5.77E-07	4.83E-07	6.35E-07
-0.160054	4.97E-07	4.43E-07	5.56E-07
-0.159666	4.95E-07	4.57E-07	5.17E-07
-0.159084	4.78E-07	4.4E-07	6.07E-07
-0.158566	5.21E-07	4.32E-07	5.38E-07
-0.15792	5E-07	4.67E-07	6.64E-07
-0.157467	4.41E-07	4.67E-07	5.33E-07
-0.157079	4.91E-07	4.78E-07	5.24E-07
-0.156432	5.21E-07	5.32E-07	5.72E-07
-0.15598	5.25E-07	4.95E-07	6.54E-07
-0.155527	5.67E-07	5.01E-07	5.77E-07
-0.155268	4.36E-07	4.61E-07	5.03E-07
-0.154622	4.56E-07	4.35E-07	4.9E-07
-0.154104	4.21E-07	4.86E-07	5.65E-07
-0.153587	4.86E-07	5.15E-07	5.65E-07
-0.153134	4.38E-07	4.27E-07	4.85E-07
-0.152488	4.99E-07	4.45E-07	5.9E-07
-0.152165	4.56E-07	4.03E-07	5.04E-07
-0.151453	4.82E-07	4.29E-07	5.36E-07
-0.151259	4.83E-07	4.25E-07	5.72E-07
-0.150677	4.5E-07	4.4E-07	5.69E-07
-0.15003	4.9E-07	4.09E-07	4.9E-07

Table S2. Normalized voltametric peak height titration data for **Figure 2B**.

Log (U/ml)	Trial 1	Trial 2	Trial 3	Trial 4	Trial 5	Trial 6
-2	0.824956	0.874251	0.987609	1.000587	0.914073	0.77525
-1.522879					0.677448	0.778637
-1	0.703343	0.628812	0.530098	0.450768		0.629979
-0.52288	0.58173	0.618368	0.34884	0.377517	0.467544	0.576763
0	0.446386	0.372929	0.331571	0.339741	0.362688	0.202744
0.477121	0.246312	0.211044	0.362284	0.367796	0.307971	0.228985
1	0.197274	0.294597	0.439599	0.463591	0.270276	0.307642

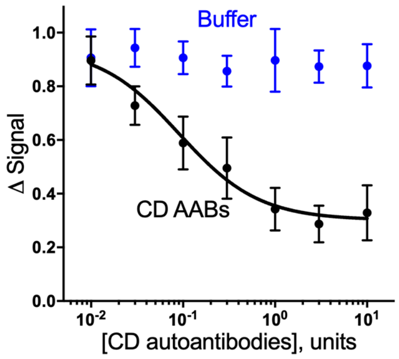

Figure S1. Biosensor response to CD AABs versus buffer control. CD AABs data (black) is reprinted from **Figure 2B**. Buffer data (blue) shows biosensor response when buffer controls were substituted for CD AABs addition.

Table S3. Normalized voltametric peak height titration in serum data for **Figure 3A**.

Log (U/ml)	Trial1	Trial 2	Trial 3
-2	0.487519		0.713
-1.522879	0.641127	0.547589	
-1	0.523896	0.59988	0.637286
-0.52288	0.465064	0.479224	
0	0.347917	0.451307	0.478143
0.477121	0.322277		
1	0.194757	0.119871	0.079492

Table S4. voltametric data for off-target analysis.

CD AABs	GADPH IgG	Myc/Max
3.2E-07	2.9E-08	8.3E-08
3.11E-07	8.88E-08	1.27E-07
2.24E-07	2.81E-08	5.44E-08
2.46E-07	8.76E-08	
6.21E-07		
1.87E-07		
